# Quadriceps muscle strength is a discriminant predictor of dependence in daily activities in nursing home residents

**DOI:** 10.1371/journal.pone.0223016

**Published:** 2019-09-24

**Authors:** Julia Wearing, Maria Stokes, Eling D. de Bruin

**Affiliations:** 1 Faculty of Health, Medicine and Sciences, School for Public Health and Primary Care, University Maastricht, Maastricht, The Netherlands; 2 School of Health Sciences, University of Southampton, Southampton, United Kingdom; 3 Institute of Human Movement Sciences and Sport (IBWS), Department of Health Sciences and Technology, ETH Zurich, Zurich, Switzerland; University of Houston, UNITED STATES

## Abstract

**Objective:**

This study aimed to explore the relationship between dependence in Activities of Daily Living and muscle strength, muscle morphology and physical function in older nursing home residents, taking possible confounders into consideration.

**Methods:**

A total of 30 nursing home residents (age, 85.6±7.1 years) were included in this observational cross-sectional study. Performance of basic Activities of Daily Living (ADL) was assessed with the Resident Assessment Instrument and categorized as either independent or dependent. Isometric grip, quadriceps and elbow-flexor strength were determined by hand-dynamometry, muscle thickness and echo intensity by B-mode ultrasonography, a sit-to-stand task by using a stop watch and physical activity by the German-Physical-Activity Questionnaire. Degree of frailty was evaluated according to Fried’s frailty criteria, whereas cognition, depression, incontinence, pain and falls were part of the Resident Assessment Instrument.

**Results:**

Dependence in Activities of Daily Living was negatively correlated with physical activity (r_s_ = -0.44, p = .015), handgrip (r_s_ = -0.38, p = .038), elbow-flexor (r_s_ = -0.42, p = .032) and quadriceps strength (r_s_ = -0.67, p < .001), analysed by Spearman’s correlation. Chronic diseases (r_s_ = -0.41, p = .027) and incontinence (r_s_ = -0.39, p = .037) were positively correlated with ADL while the other variables were not related. Only quadriceps strength remained significant with logistic regression (Wald(1) = 4.7, p = .03), when chronic diseases, quadriceps and handgrip strength were considered (R^2^ .79). 11 kg was the best fitting value in this sample to predict performance in Activities of Daily Living, evaluated with Receiver-Operating Characteristic analysis, with a sensitivity of 100% and a specificity of 79%.

**Conclusion and implication:**

Quadriceps strength had a positive independent relationship with performance in ADL in the nursing home residents studied. Although a large prospective study is needed to verify the results, maintaining quadriceps strength above 11 kg may be helpful in retaining independence in this cohort.

## Introduction

In 2017, 15.7% of the Swiss population aged 80 years and over were institutionalized [1]. The demand for long-term care is expected to expand [2] since life expectancy will continue to increase in industrialized countries [3], with an expected rise of the old-old population by 300% in 2050 [4]. Admission of older persons to nursing homes is dependent on cognitive and/or functional impairment in combination with a lack of assistance in daily home life, which often leads to dependence in activities of daily living (ADL) [5]. In 30–50% of these people, dependence actually increases within the first 18 months of institutionalization due to further functional decline [6, 7], which adversely affects quality of life [8] and health care costs [7]. Prevention of physical decline in nursing home residents is, therefore, essential to maintain a certain amount of independence, with beneficial effects for the individual as well as for the health care system.

The ability of nursing home residents to perform ADL independently is associated with multiple factors, both modifiable and non-modifiable, but is mainly dependent on age, chronic disease and disability, with the latter factor being the most discriminant predictor [9]. Physical disability in old age is highly associated with low muscle strength, which decreases progressively due to an age-related decline in muscle mass and quality [10]. Muscle strength is reduced by up to 50% in people aged 80 years and over [11], with highest rates of loss in physically inactive individuals [12, 13] that are institutionalized in nursing homes [14].

Previous studies involving community dwelling older people have shown the relevance of quadriceps strength for e.g. independent performance of sit-to-stand tasks [15, 16] and the effortless execution of ADL [15, 17]. In older people in need of long-term care, only a few studies investigated whether quadriceps strength relates to the level of required care and findings were inconclusive [18–20]. A positive association between quadriceps strength and ADL performance was confirmed by intervention studies that have shown training to be effective in improving physical function even in non-healthy, non-robust, ADL-dependent older adults suffering from disuse-related muscle weakness [21–26]. However, the optimal program remains unclear [23]. Therefore, specifying the underlying physical determinants of dependence in basic ADL of institutionalized older adults would help to determine the most important component of a specific training program.

In the present study, we aimed to investigate whether muscle structure, strength, function or physical activity were predictive variables of dependence in ADL in nursing home residents, when accounting for cognition, depression, falls, incontinence, chronic disease, sedative medication and pain.

## Methods

### Study design

A cross-sectional study of muscle characteristics and physical function of nursing home residents was undertaken in a long-term care institution in Switzerland between August and December 2017. Recruitment targeted older adults, aged 65 years and over. Exclusion criteria were a) an inability to understand study content and provide sign informed consent; b) severely impaired decision making (Cognitive performance scale > 4 points) [27]; c) a history of acute lower limb pathology (fracture and/or surgery within the last 6 months); d) skin disorders involving the anterior thigh and/or arm; e) limb paralysis; and f) confinement to bed.

Nursing home residents were screened by the senior nurse for exclusion criteria based on the last RAI assessment. Only participants who were deemed competent to consent and voluntarily agreed to participate were considered for study inclusion. All study procedures complied with the principles of the Declaration of Helsinki for ethical research in humans and the study received approval from the local ethics committee (project-ID 2017-00839).

Details are presented in the following report, which adheres to the reporting guidelines for cross-sectional studies [28].

### Sample size

Sample size was calculated a priori using published data for quadriceps strength in nursing home residents [20]. A total sample size of 19 participants was required to ensure sufficient statistical power (β = 0.2) to detect a 25% difference in strength between ADL independent and dependent groups (α = .05). It has been previously shown that disabled older adults, as defined by self-reported independence in basic and instrumental ADL, exhibit up to 50% lower muscle strength than non-disabled individuals [17].

### Data collection

#### Participant demographics

Participant demographics, medications history, medical history and independence in ADL were obtained using of the Long-Term Care Facility Resident Assessment Instrument (RAI; Minimum Data Set Version 2.0). For assessment of ADL, urinary incontinence, cognitive performance and depressive symptoms, observations were judged and encoded by one of four nurses who were experienced RAI-item coders. All nurses had received the same training on the use of the tool in its application. Detailed RAI items are shown in Table 1.

**Table 1:** Items of the Resident Assessment Instrument obtained from the study participants.

RAI items	Units and classification
age	years
height	meters
weight	kilogram
urinary continence	4-point-scale from 0 (= continent) to 4 (= always incontinent, no bladder control)
pain intensity	Numeric Analog Scale from 0 (= no pain) to 10 (= worst pain)
falls	frequency within last three months
cognitive performance	Minimum Data Set Cognitive Performance Scale (scale ranging from 0 (= intact cognition) to 6 (= severely limited cognition)
frequency of depressive symptoms	scale from 0 (symptoms weren’t shown) to 2 (symptoms shown on 6/7 days/week)
amount and type of chronic diseases	metabolic, musculoskeletal, neurological, psychiatric, respiratory disease, renal insufficiency, vertigo and cancer
regular intake of medication	antidepressants, sedatives and muscle relaxants
self-performance in activities of daily living (ADL)	bed mobility, transfer, walking in a room, walking in a corridor, locomotion on the ward, locomotion outside the ward, dressing, eating/drinking, toilet use and personal hygiene over the past 7 days, each rated on a scale from 0 (independent) to 4 (fully dependent), full range of possible outcome 0–40. Categorization as independent in ADL when total score = 0, which reflected no need for assistance or staff oversight. Categorization as dependent when total score ≥ 1, which reflected a need for assistance or staff oversight in at least one activity

One investigator, a physiotherapist, trained and experienced in musculoskeletal assessments and ultrasonography, completed the following test series for all participants.

#### Muscle strength

Maximal isometric quadriceps and elbow-flexor muscle strength, as the highest of two trials, was evaluated using a hand-held dynamometer (Microfet2^®^, CompuFET, Hoggan Health Industries, Biometrics Europe). For measurement of elbow-flexor strength, the participant was seated on a chair, elbow flexed at 90°and forearm supinated. For measurement of quadriceps strength, the participant was seated on a plinth, with their back resting against a firm support, thighs fully supported, knees flexed to 90° and the lower legs hanging freely. The curved transducer pad of a hand-held dynamometer was positioned at 80% of the forearm and tibial length respectively, to resist maximal isometric force of the elbow flexors and quadriceps. The participants were asked to push against the dynamometer as hard as possible for 3 seconds. Strength was measured in Newtons (N), and converted into Kilograms (kg) by dividing N by 9.81. Torque was calculated in Newtonmeters (Nm) by multiplying force by the lever arm length of the forearm and tibia respectively. Hand-held dynamometry has been shown to be a valid and reliable technique to assess isometric strength in older adults [29, 30]. Test-retest reliability of hand-held dynamometry is high when assessed by a trained examiner using a standardized protocol [31]; with intra-rater reliability Intraclass Correlation Coefficient (ICC) ranging between 0.90 and 0.98 [29].

Handgrip strength was measured with a hand dynamometer (Jamar^®^, Lafayette, USA) according to the standardized protocol recommended by the American Society of hand therapists [32].

#### Muscle morphology

Real-time, B-mode ultrasonography (Nemio MX Type SSA-590A, Toshiba, Japan) with a 12 MHz linear transducer array (45 mm footprint) was used to obtain transverse images of the dominant extremities. Images of the rectus femoris/vastus intermedius as well as the biceps brachii/brachialis were taken using a previously published protocol for community-dwelling older adults [33, 34], and post-processed using semi-automated MATLAB code (MathWorks^®^, Massachusetts, USA). Muscle thickness and primary muscle echotexture statistics were subsequently calculated, with the mean of two images taken for further analysis.

The thickness of the muscles was defined as the distance between the inner border of the fascial layer that distinguishes muscles from superficial fat and bone. Ultrasound-based measures of thigh tissue thickness are highly correlated with the gold standard of Magnetic Resonance Imaging (r = 0.99) [35] and have a reported intra-rater reliability of ICC 0.88-.099 in older people [33, 36].

For grayscale analysis, settings of the ultrasound scanner (gain, time gain control, dynamic range value, focus and power) were adjusted to assure good quality of the images and kept constant for all participants; depth was readjusted to individual muscle thickness. Echogenicity of rectus femoris and biceps brachii was defined as the average grayscale within a rectangular region of interest and recorded as unspecified units (UU) 0–255. The analysis method has been previously shown to have high intra-rater reliability, with a reported ICC of 0.97–0.99 [37]. High muscle echo intensity is associated with low tissue density in CT scans [38] and high adipose tissue content in muscle biopsies [39].

#### Functional mobility

Functional mobility was estimated using the sit-to-stand task repeated 5 times [40]. A chair with a straight backrest, 40 cm seat height and without armrests was placed against a solid support. Participants were instructed to complete 5 sit-to-stand maneuvers as fast as possible without the use of their arms. The time taken for completion was recorded. Times in excess of 13.6 seconds are associated with increased disability [41].

#### Physical activity

The German physical activity 50+ questionnaire was used to calculate energy expenditure (kcal/week) [42]. The questionnaire has a test-retest reliability of r = 0.52–0.6 [42] and is widely used in German speaking countries to evaluate physical activity in older people.

#### Frailty

Physical frailty was evaluated according to Fried’s frailty criteria [43]. Participants were characterized as “not frail”, “pre-frail” or “frail” according to the number of positive criteria identified (0, 1–2 and ≥ 3, respectively).

### Statistical analysis

The Statistical Package for the Social Sciences (SPSS Statistics, Version 23.0. IBM Corporation, Armonk, NY) was used for analysis. Shapiro-Wilk test was used to test for normality. Dependent on data distribution, descriptive statistics are presented as mean ± standard deviation or median (range); differences between the ADL groups were analyzed using independent t-tests and Mann-Whitney-U-test. Relationships of ADL performance with independent variables were identified by Spearman’s correlation coefficients and binary logistic regression analysis. Receiver-Operating Characteristic (ROC) curve was utilized for sensitivity/specificity analysis of predictors of ADL performance.

## Results

### Characteristics of study population

Of 177 nursing home residents who were screened, 30 fulfilled the eligibility criteria and were included in the study (mean age (SD) 85.7 (7.1) years, 76.7% female). 43% of the study sample were categorized as pre-frail, 57% as frail, 97% had ≥ two chronic diseases, 30% experienced depressive symptoms, 20% took sedative medication, 62% were cognitively impaired, while 70% were diagnosed with dementia, 23% had fallen, 34% experienced pain and 28% were incontinent. Four participants were excluded from strength measurements due to concerns associated with the risk of osteoporotic fracture and 14 were incapable of rising from a chair without using their arms. Given that only half of the participants could rise from a chair 5 times without using their arms, functional mobility was dichotomized into the categories “able to complete 5STS” and “unable to complete 5STS” for further analysis.

**Fig 1:**
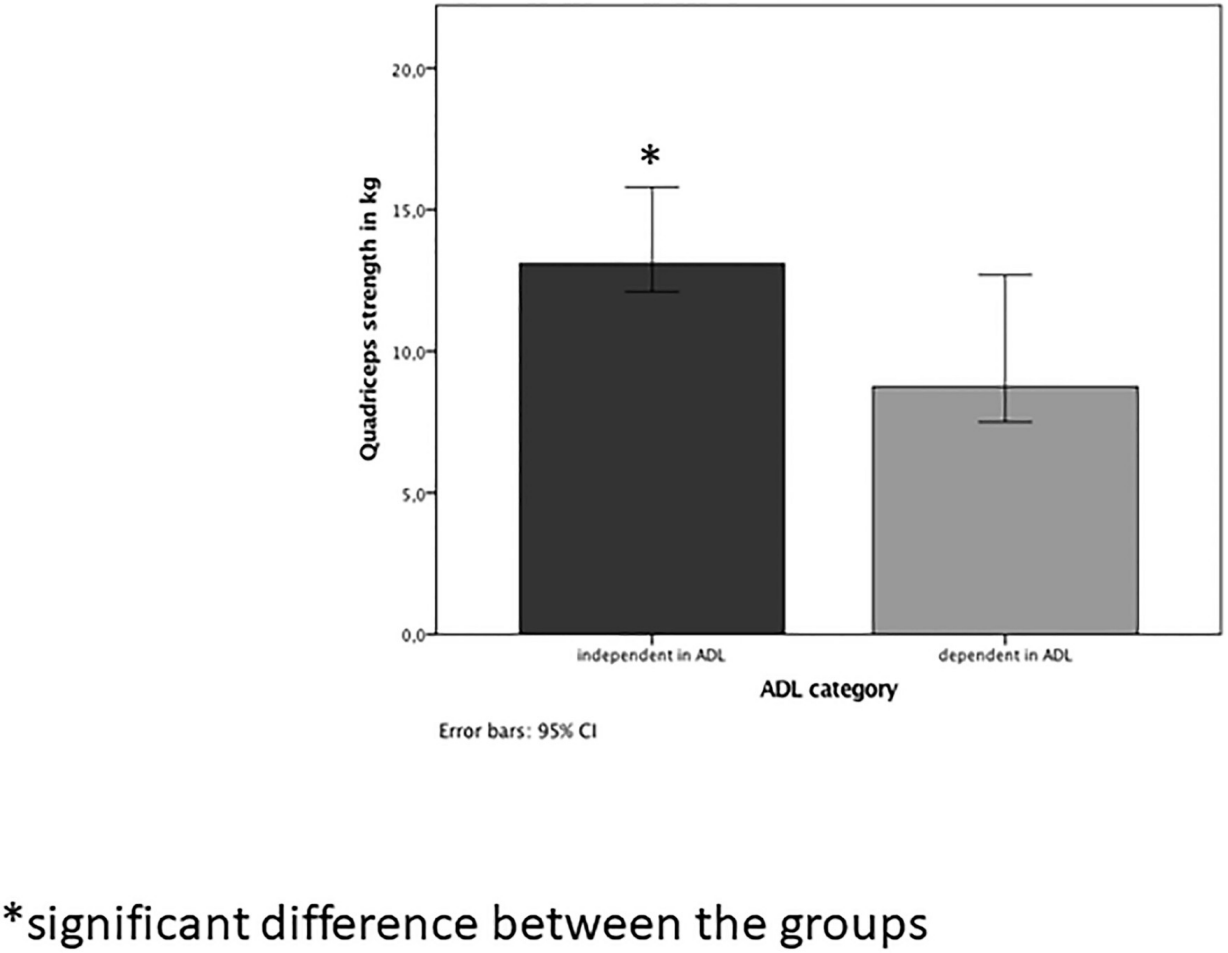
Quadriceps strength in people independent and dependent in ADL.

Of the 30 participants, 16 were categorized as independent and 14 as dependent in ADL based on items of the RAI. There were no statistically significant differences between groups in terms of age, weight, height, number of years institutionalized, medication history, as well as for muscle morphology, functional mobility and physical activity. The group dependent in ADL experienced a significantly higher number of chronic diseases and more severe incontinence, lower handgrip, elbow-flexor and quadriceps strength. Quadriceps strength of participants independent in ADL was greater by a median of 4.3 kg than those dependent in ADL (Fig 1).

Characteristics of participants are presented in Table 2.

**Table 2:** Participants’ characteristics of the ADL dependent and independent group.

Characteristic (unit)	ADL independent	ADL dependent	equality of means/medians	
	Mean (SD)/Median (range)	Mean (SD)/Median (range)	t/U	p
**Demographics**				
*Age*^†^ *(years)*	87.0 (15)	86.0 (35)	U = 100.5	.64
*Weight*^†^ *(kg)*	66.5 (52.2)	68.0 (51)	U = 98.0	.58
*Height*^†^ *(m)*	1.64 (0.23)	1.59 (0.21)	U = 80.5	.19
*Incontinence (0–4 points)*	0 (1)	0 (4)	U = 68	.038*
*Pain intensity (0–10)*	0.53 (0.74)	0.64 (1.60)	t(18.1) = -.234	.82
*Falls (number)*	0.63 (1.63)	0.29 (0.47)	t(17.8) = .797	.44
*Cognitive performance (0–6)*	1.0 (3)	2.0 (3)	U = 79	.24
*Depressive symptoms (0–32)*	1.62 (3.95)	0.71 (1.27)	t(25) = 0.815	.42
*Chronic diseases (number)*	2.75 (1.24)	3.86 (1.29)	t(27.1) = -2.387	.024*
*Medication (number)*	0.81 (0.40)	0.79 (0.43)	t(27.0) = .176	.86
*Institutionalization (years)*	2.9 (2.2)	3.4 (1.8)	t(27.8) = -.572	.57
**Muscle strength**				
*Handgrip strength*^†^ *(kg)*	16.0 (22)	13.3 (13)	U = 63.0	.043*
*Quadriceps strength*^†^ *(kg)*	13.1 (5.7)	8.8 (6.9)	U = 18.5	<.001*
*Quadriceps strength*^†^ *(N)*	128.2 (56.4)	85.4 (63.3)	U = 18.5	<.001*
*Strength/body weight (N/kg)*	2.1 (0.7)	1.4 (0.4)	t(16.7) = 2.797	.013*
*Quadriceps torque*^†^ *(Nm)*	39.1 (18.8)	26.8 (20.3)	U = 20	.001*
*Torque/body weight (Nm/kg)*	0.6 (0.2)	0.4 (0.1)	t(16.9) = 2.763	.013*
*Elbow-flexor strength*^†^ *(kg)*	9.7 (7.3)	7.0 (9.5)	U = 43.0	.036*
**Muscle morphology**				
*Quadriceps thickness (mm)*	17.7 (4.7)	18.7 (6.7)	t(28) = -.465	.65
*Rectus femoris grayscale*^†^ *(UU)*	117.2 (78)	115.5 (101)	U = 109.0	.92
*Elbow-flexor thickness*^†^ *(mm)*	24.8 (18)	26.5 (16)	U = 111	.98
*Biceps brachii grayscale*^†^ *(UU)*	132 (81)	133 (86)	U = 111	.98
**Functional mobility**				
*5 sit-to-stand (sec)*	20.3 (11.6)	20.6 (12.3)	t(8.0) = -.051	.96
**Physical activity (kcal/week)**	842 (879)	508 (1347)	t(21.9) = .792	.44

* significant difference

^†^ data not normally distributed, differences determined with the Mann-Whitney-U test, p equates exact significance

Quadriceps strength, in the present study, was not related to body weight. Therefore, actual strength values in kg were used for analysis. To aid comparison with previous research, quadriceps strength was also expressed as a ratio of strength [(N)/body weight (kg)].

Of the group that was independent in ADL (n = 16), 9 participants could complete the 5STS, while 7 could not. Of the group that was dependent in ADL (n = 14), 5 participants could complete the 5STS, while 9 were not able to complete the 5STS.

### Correlations of ADL category with demographics, medical and medications history

The level of ADL dependence significantly correlated with a higher number of comorbidities (r_s_ = .41, p = .027) and incontinence (r_s_ = .39, p = .037), but was not associated with sex, age, falls, cognitive performance, sedative medication, depressive mood, pain or physical frailty at the univariate level (S1 Appendix).

### Correlations of ADL category with muscle strength, muscle morphology, functional mobility and physical activity

Dependence in ADL was weakly associated with lower handgrip strength (r_s_ = -0.38, p = .038), lower elbow-flexor strength (r_s_ = -0.42, p = .032) and lower physical activity (r_s_ = -0.44, p = .015) but moderately correlated with lower quadriceps strength (r_s_ = -0.67, p < .001). There was no correlation of ADL dependence with muscle morphology and functional mobility/the ability to rise from a chair 5 times (S2 Appendix).

### Regression and ROC analysis

To determine factors that were predictive of ADL performance, binary logistic regression was undertaken iteratively using different combinations of covariates, including the factor with the highest correlation with ADL (quadriceps strength) and factors that were moderately correlated with ADL (handgrip strength, elbow-flexor strength, urinary incontinence and number of chronic diseases) at the univariate level (S1 and S2 Appendices).

The combination of variables that best explained the variance in ADL performance included quadriceps strength, chronic diseases and handgrip strength (S3 Appendix). Approximately 79% of the variance was explained by these three factors (Nagelkerke’s R^2^ = 0.786). However, quadriceps strength was the only independent predictor of dependence with an Odds Ratio (OR) of 0.353 (95% CI 0.138–0.905, p = .030), indicating a 65% lower risk of being dependent when quadriceps strength increased by 1 kg (Table 3).

**Table 3:** Binary logistic regression with inclusion analysis of the variables quadriceps strength, chronic diseases and physical activity.

predictor	Regression coefficient (B)	Significance level (p)	Exp(B) = OR	95% CI for Exp(B)	
				lower	upper
quadriceps strength	-1.040	.030	0.353	0.138	0.905
chronic diseases	1.325	.074	3.763	0.877	16.139
handgrip strength	-.213	.390	0.809	0.498	1.313
constant	10.863	.067	52183.975		

The ROC curve for analysis of sensitivity and specificity of quadriceps strength to identify people independent and dependent in ADL showed an area under the curve of 0.89. Strength of 11.25 kg was the best fitting value with a sensitivity of 100% and a specificity of 79%. Sensitivity and specificity of quadriceps strength values are shown in Table 4.

**Table 4:** Coordinates of ROC curve analysis for accuracy of quadriceps strength in detecting residents of nursing homes dependent in ADL.

Quadriceps strength (kg) greater than or equal to	Sensitivity	1-specificity
5.800	1.000	1.000
7.000	1.000	.929
7.350	1.000	.857
7.550	1.000	.786
7.900	1.000	.643
8.450	1.000	.571
8.750	1.000	.500
9.300	1.000	.429
10.150	1.000	.357
10.600	1.000	.286
^‡^11.250	1.000	.214
11.950	.833	.214
12.150	.750	.214
12.300	.667	.214
12.550	.583	.214
12.850	.583	.143
13.100	.500	.071
13.350	.417	.071
13.600	.333	.071
13.900	.333	0.000
14.950	.250	0.000
16.650	.083	0.000
18.500	0.000	0.000

^‡^ Best fitting quadriceps strength value to detect dependent/independent performance of ADL

## Discussion

This study aimed to evaluate potential muscle-related predictors of dependence in ADL in nursing homes residents, taking cognitive function, depression, pain, urinary incontinence, chronic diseases, medication and falls into consideration.

Of the investigated parameters, greater handgrip-strength, elbow-flexor strength, quadriceps strength and physical activity, as well as less incontinence and chronic diseases, were positively associated with the ability to independently perform basic ADL whereas quadriceps strength was the only independent predictor.

The positive relationship observed between quadriceps strength and ADL performance is consistent with a previous study in which functional performance was evaluated in older people with dementia in need of long-term care [20]. ADL-independent participants in their study had 40–45% higher strength than ADL-dependent, while ours differed by about 33%. Suzuki and colleagues specifically included people diagnosed with dementia, whereas the present study also included participants without dementia and of different cognitive performance levels. Interestingly, neither quadriceps strength nor ADL dependence was related to cognitive performance in our population. This is opposed to previously reported findings [44] and might be due to the small size of our sample.

The present findings do not correspond with other studies in which the level of care was observed to be independent of quadriceps strength in old, physically disabled people [18, 19]. These earlier studies differed in regard to the assessment instrument (care time [19], respectively a non-specified instrument [18] versus RAI), included ADL (basic and instrumental ADL [19] respectively non-specified assessment [18] versus basic ADL) and categorization of ADL performance (3-point scale [18, 19] versus dichotomous classification). However, the main difference is potentially the type of ADL on which the categorization is based. In contrast to most basic activities, many instrumental ADL, such as the regulation of finances and telephone use, do not require appreciable quadriceps strength. Hence, it could be expected that when ADL dependence is categorized on the basis of instrumental activities, it is unlikely to be correlated with quadriceps strength.

Consequently, the present study adds the following new information to previous findings: 1. the relationship between low quadriceps strength and ADL dependence may be valid for residents in nursing homes independent of cognitive performance; 2. quadriceps strength not only has a significant relation to dependence in basic ADL but also has a strong independent association with ADL dependence regardless of muscle structure, muscle function, physical activity and important confounding factors of demographics, medical and medication history.

It is an interesting outcome that low quadriceps strength but not low muscle thickness or high echo intensity was associated with ADL dependence. One possible explanation for this finding could be that a decline in motor cortical properties rather than changes in muscle morphology accounted for low voluntary strength in our population. Muscle weakness associated with aging has diverse underlying mechanisms and is not solely explained by atrophy of muscle [45]. The nervous system’s overall ability to maximally activate a muscle, including descending drive from the motor cortex, also declines with age and significantly contributes to decreased voluntary contraction of available musculature [46]. Particularly in older-old individuals, voluntary activation, defined as “the level of voluntary drive during an effort” [47, 48] is diminished [49–51] and may account for up to one third of the loss in force production [52].

Furthermore, it seems contradictory that an older person may have adequate quadriceps strength to rise from a chair independently but is not able to complete the repeated sit-to-stand exercise (5STS). However, while poor performance on the STS test (>10–13 seconds) has been shown to predict incidence of disability in older community-living older adults [40, 53], its sensitivity is limited (50%) and its clinical use could be largely restricted to high functioning, community-living older people [54]. Quadriceps strength is one important underlying precondition of the ability to stand up from a chair [55], however, it is not the only determinant. In most elderly nursing home residents the ability to rise from a standard-height chair is also dependent on the use of arms and arm rests [56] and, when not constrained by artificially imposed time limits, may still be performed independently. Completion of the 5STS test, in comparison, requires more complex abilities, as it is performed without the use of arms and is timed. Hence, the 5STS test needs coordinated contraction and high contraction speed (power) of multiple lower extremity muscles, including the gluteal and ankle dorsi flexor muscles [57–59]. Secondly, successful completion of 5STS also requires balance, proprioception and tactile sensation [58], all of which also decrease with age and disease. Thirdly, voluntary functional strength in older people might be mainly explained by reduced voluntary activation due to changes in central and peripheral nervous systems [46]. The functional mobility task of 5STS with its physical requirements, therefore, may not reflect the abilities necessary to live an ADL independent, nursing home life. Rather, quadriceps strength would appear to be a better predictor of ADL disability than the 5STS test in this population of lesser functioning older people.

An isometric quadriceps strength of > 11kg predicted ADL-independence by 100% and ADL-dependence by 79% in this study sample. The findings show that quadriceps strength was of high importance for explaining the variance in ADL performance; an improvement of 1 kg lowers the risk of becoming dependent by 65%. Interventional studies have shown that exercise programs of 8–12 weeks, including resistance exercises 2–3 times weekly, were effective in increasing quadriceps strength in older, nursing home residents by a minimum of 3 kg [18, 22]. Thus, nursing home residents with physical disability may be able to improve ADL independence through quadriceps strengthening. This finding supports the call for preventive measures to avoid functional decline in nursing home dwellers [7].

Previous research has established that quadriceps strength differs significantly in community living older people independent in ADL (3.5–3.8 N/kg) from those partially dependent (2.2–2.9 N/kg) [15], when independence is considered as being able to e.g. walk 50 m without any personal or device support and without loss of normal speed and safety. In comparison, the present study showed that frail, disabled, nursing home residents of the same age were only half as strong, with the independent group having a median strength of 2.1 N/kg, compared to the ADL dependent group with median strength of 1.4 N/kg. However, approximately half of the participants could still perform usual nursing-home ADL, such as walking a few meters with a walker and rising from a chair with the use of arm rests, without supervision or assistance of another person.

Some limitations of this cross-sectional study should be mentioned. Firstly, observations of behavior and emotions by nursing staff entailed the risk that some actions of the participants could have been unrecognized and therefore not recorded adequately. However, the observation period included seven days in which residents were closely observed by a trained nurse attentive to precise assessment. Therefore, the risk of information bias was likely to be small. Even though RAI data were obtained by different assessors, data can still be assumed sufficiently reliable since inter-rater reliability of the RAI items has been shown to be 0.63–0.92 (weighted Kappa) [60]. Secondly, physical activity measures were based on self-report and behavioral observations of nursing staff. Although previous studies have questioned the accuracy of self-reported measures of physical activity in older populations [61], participants were closely monitored during the study so that any discrepancy between reported and actual activity was likely to be small. Thirdly, the number of participants who could complete the timed sit-to-stand task was rather small. Findings with regard to this variable could therefore be underpowered. However, even when the participants were categorized into two groups depending on their ability to complete the task, results did not change. Therefore, the results were assumed to be valid for this cohort. Fourthly, a causal relationship between ADL performance and quadriceps strength cannot be made due to the nature of the cross-sectional study design. However, the results indicate a strong association between ADL dependence and low quadriceps strength and longitudinal studies have demonstrated beneficial effects of quadriceps’ strength training on physical function [21–26]. Fifthly, the study sample only included participants from one nursing home. Therefore, the results of the sample might not be generalizable to a wider population of older, frail nursing home residents. The study did, however, include a wide variety of participants with regard to ADL performance. Therefore, the participants could be considered representative of the target population.

## Conclusions

This study has shown that strength, physical activity and incontinence were potentially modifiable factors associated with ADL dependence in nursing home residents, with quadriceps strength being the only independent predictor of dependence in ADL, independent of age, frailty status, co-morbidities and cognitive function.

Although further research is required, interventions aimed at increasing these physical abilities with a specific focus on enhancing leg muscle strength beyond target threshold values may be a useful strategy for reducing dependence in ADL of nursing home dwellers.

## Supporting information

S1 Appendix Correlations of ADL category with demographics. (DOCX)

S2 Appendix Correlations of ADL category with muscle-related parameter. (DOCX)

S3 Appendix Binary logistic regression. (DOCX)

S4 Appendix STROBE statement—Checklist. (DOCX)
