# Supplementary material for: Quadriceps muscle strength is a discriminant predictor of dependence in daily activities in nursing home residents
Source: PLoS One. 2019 Sep 24;14(9):e0223016. doi: 10.1371/journal.pone.0223016 (PMC6759157; doi:10.1371/journal.pone.0223016)
Supplement: S1 Appendix — (DOCX) [file pone.0223016.s001.docx]

S1 Appendix: Correlations of ADL category with demographics

| variable | Correlation coefficient (r_s_) | Significance (p) |
| --- | --- | --- |
| demographics |  |  |
| age | -.09 | .639 |
| sex | -.12 | .542 |
| height | -.24 | .194 |
| weight | .11 | .570 |
| urinary incontinence | .39 | .037* |
| pain intensity | -.12 | .543 |
| falls | .07 | .701 |
| cognitive performance | .23 | .238 |
| frequency of depressive symptoms | .09 | .660 |
| symptoms of physical frailty | .22 | .234 |
| amount of chronic diseases | .41 | .027* |
| regular intake of medication | -.03 | .861 |

*significant difference
