# Supplementary material for: Quadriceps muscle strength is a discriminant predictor of dependence in daily activities in nursing home residents
Source: PLoS One. 2019 Sep 24;14(9):e0223016. doi: 10.1371/journal.pone.0223016 (PMC6759157; doi:10.1371/journal.pone.0223016)
Supplement: S2 Appendix — (DOCX) [file pone.0223016.s002.docx]

S2 Appendix: Correlations of ADL category with muscle-related parameter

| variable | Correlation coefficient (r_s_) | Significance (p) |
| --- | --- | --- |
| muscle strength |  |  |
| handgrip strength | -0.38 | =.038* |
| elbow flexor strength | -0.42 | =.032* |
| quadriceps strength | -0.67 | <.001* |
| muscle morphology |  |  |
| quadriceps thickness | .01 | =.968 |
| rectus femoris grayscale | -.02 | =.903 |
| elbow-flexor thickness | -.01 | =.968 |
| biceps brachii grayscale | .01 | =.968 |
| functional mobility/ 5 sit-to-stand category | -.21 | =.276 |
| physical activity | -0.44 | =.015* |

*significant difference
