# Supplementary material for: Quadriceps muscle strength is a discriminant predictor of dependence in daily activities in nursing home residents
Source: PLoS One. 2019 Sep 24;14(9):e0223016. doi: 10.1371/journal.pone.0223016 (PMC6759157; doi:10.1371/journal.pone.0223016)
Supplement: S3 Appendix — (DOCX) [file pone.0223016.s003.docx]

S3 Appendix: Binary logistic regression

| predictor | Regression coefficient (B) | Significance level (p) | Exp(B)=OR | 95% CI for Exp(B) |  |
| --- | --- | --- | --- | --- | --- |
|  |  |  |  | lower | upper |
| Quadriceps strength | -.992 | .014 | 0.371 | 0.167 | 0.821 |
| constant | 11.782 | .017 | 130829.640 |  |  |

Nagelkerke’s R^2^=0.65

| predictor | Regression coefficient (B) | Significance level (p) | Exp(B)=OR | 95% CI for Exp(B) |  |
| --- | --- | --- | --- | --- | --- |
|  |  |  |  | lower | upper |
| Quadriceps strength | -1.166 | .031 | 0.312 | 0.108 | 0.901 |
| Incontinence category | 1.957 | .174 | 7.078 | 0.422 | 118.848 |
| constant | 13.251 | .038 | 568610.962 |  |  |

Nagelkerke’s R^2^=0.69

| predictor | Regression coefficient (B) | Significance level (p) | Exp(B)=OR | 95% CI for Exp(B) |  |
| --- | --- | --- | --- | --- | --- |
|  |  |  |  | lower | upper |
| Quadriceps strength | -1.128 | .018 | 0.324 | 0.127 | 0.825 |
| Chronic diseases | 1.092 | .091 | 2.979 | 0.839 | 10.579 |
| constant | 9.575 | .083 | 14403.725 |  |  |

Nagelkerke’s R^2^=0.77

| predictor | Regression coefficient (B) | Significance level (p) | Exp(B)=OR | 95% CI for Exp(B) |  |
| --- | --- | --- | --- | --- | --- |
|  |  |  |  | lower | upper |
| Quadriceps strength | -.992 | .053 | 0.317 | 0.136 | 1.012 |
| Chronic diseases | 1.091 | .090 | 2.979 | 0.845 | 10.504 |
| Elbow-flexor strength | -.205 | .591 | 0.815 | 0.387 | 1.717 |
| constant | 10.483 | .087 | 19426.475 |  |  |

Nagelkerke’s R^2^=0.78

| predictor | Regression coefficient (B) | Significance level (p) | Exp(B)=OR | 95% CI for Exp(B) |  |
| --- | --- | --- | --- | --- | --- |
|  |  |  |  | lower | upper |
| Quadriceps strength | -1.127 | .019 | 0.324 | 0.126 | 0.832 |
| Chronic diseases | .998 | .149 | 2.712 | 0.699 | 10.528 |
| Incontinence category | .709 | .607 | 2.203 | 0.108 | 44.774 |
| constant | 9.614 | .094 | 14967.836 |  |  |

Nagelkerke’s R^2^=0.76

| predictor | Regression coefficient (B) | Significance level (p) | Exp(B)=OR | 95% CI for Exp(B) |  |
| --- | --- | --- | --- | --- | --- |
|  |  |  |  | lower | upper |
| Quadriceps strength | -1.149 | .019 | 0.317 | 0.121 | 0.828 |
| Chronic diseases | .976 | .126 | 2.655 | 0.761 | 9.262 |
| Physical activity | .000 | .572 | 1.000 | 0.998 | 1.001 |
| constant | 10.483 | .089 | 35706.192 |  |  |

Nagelkerke’s R^2^=0.77
