# Supplementary material for: Quadriceps muscle strength is a discriminant predictor of dependence in daily activities in nursing home residents
Source: PLoS One. 2019 Sep 24;14(9):e0223016. doi: 10.1371/journal.pone.0223016 (PMC6759157; doi:10.1371/journal.pone.0223016)
Supplement: S4 Appendix — (DOCX) [file pone.0223016.s004.docx]

STROBE Statement—checklist

|  | | Item No. | | Recommendation | | Page  No. | | | Relevant text from manuscript | | |
| --- | --- | --- | --- | --- | --- | --- | --- | --- | --- | --- | --- |
| **Title and abstract** | | 1 | | (*a*) Indicate the study’s design with a commonly used term in the title or the abstract | | 2 | | | A total of 30 nursing home residents (age, 85.6±7.1 years) were included in the observational cross-sectional study. | | |
|  |  |  |  | (*b*) Provide in the abstract an informative and balanced summary of what was done and what was found | | 2/3 | | | Objective: This study aimed to explore the relationship between dependence in Activities of Daily Living and muscle strength, muscle morphology and physical function in older nursing home residents, taking possible confounders into consideration.  Methods: A total of 30 nursing home residents (age, 85.6±7.1 years) were included in this observational cross-sectional study. Performance of basic Activities of Daily Living (ADL) was assessed with the Resident Assessment Instrument and categorized as either independent or dependent. Isometric grip, quadriceps and elbow-flexor strength were determined by hand-dynamometry, muscle thickness and echo intensity by B-mode ultrasonography, a sit-to-stand task by using a stop watch and physical activity by the German-Physical-Activity Questionnaire. Degree of frailty was evaluated according to Fried’s frailty criteria, whereas cognition, depression, incontinence, pain and falls were part of the Resident Assessment Instrument.  Results: Dependence in Activities of Daily Living was negatively correlated with physical activity (rs=-0.44, p=.015), handgrip (rs=-0.38, p=.038), elbow-flexor (rs=-0.42, p=.032) and quadriceps strength (rs=-0.67, p<.001), analysed by Spearman’s correlation. Chronic diseases (rs=-0.41, p=.027) and incontinence (rs=-0.39, p=.037) were positively correlated with ADL while the other variables were not related. Only quadriceps strength remained significant with logistic regression (Wald(1)=4.7, p=.03), when chronic diseases, quadriceps and handgrip strength were considered (R2 .79). 11 kg was the best fitting value in this sample to predict performance in Activities of Daily Living, evaluated with Receiver-Operating Characteristic analysis, with a sensitivity of 100% and a specificity of 79%.  Conclusion and implication: Quadriceps strength had a positive independent relationship with performance in ADL in the nursing home residents studied. Although a large prospective study is needed to verify the results, maintaining quadriceps strength above 11 kg may be helpful in retaining independence in this cohort. | | |
| Introduction | | | | | | | |  | | |  |
| Background/rationale | | 2 | | Explain the scientific background and rationale for the investigation being reported | | 4/5 | | | In 2017, 15.7% of the Swiss population aged 80 years and over were institutionalized [1]. The demand for long-term care is expected to expand [2] since life expectancy will continue to increase in industrialized countries [3], with an expected rise of the old-old population by 300% in 2050 [4]. Admission of older persons to nursing homes is dependent on cognitive and/or functional impairment in combination with a lack of assistance in daily home life, which often leads to dependence in activities of daily living (ADL) [5]. In 30-50% of these people, dependence actually increases within the first 18 months of institutionalization due to further functional decline [6, 7], which adversely affects quality of life [8] and health care costs [7]. Prevention of physical decline in nursing home residents is, therefore, essential to maintain a certain amount of independence, with beneficial effects for the individual as well as for the health care system.  The ability of nursing home residents to perform ADL independently is associated with multiple factors, both modifiable and non-modifiable, but is mainly dependent on age, chronic disease and disability, with the latter factor being the most discriminant predictor [9]. Physical disability in old age is highly associated with low muscle strength, which decreases progressively due to an age-related decline in muscle mass and quality [10]. Muscle strength is reduced by up to 50% in people aged 80 years and over [11], with highest rates of loss in physically inactive individuals [12, 13] that are institutionalized in nursing homes [14].  Previous studies involving community dwelling older people have shown the relevance of quadriceps strength for e.g. independent performance of sit-to-stand tasks [15, 16] and the effortless execution of ADL [15, 17]. In older people in need of long-term care, only a few studies investigated whether quadriceps strength relates to the level of required care and findings were inconclusive [18-20]. A positive association between quadriceps strength and ADL performance was confirmed by intervention studies that have shown training to be effective in improving physical function even in non-healthy, non-robust, ADL-dependent older adults suffering from disuse-related muscle weakness [21-26]. However, the optimal program remains unclear [23]. Therefore, specifying the underlying physical determinants of dependence in basic ADL of institutionalized older adults would help to determine the most important component of a specific training program. | | |
| Objectives | | 3 | | State specific objectives, including any prespecified hypotheses | | 5 | | | In the present study, we aimed to investigate whether muscle structure, strength, function or physical activity was a predictive variable of dependence in ADL in nursing home residents, when accounting for cognition, depression, falls, incontinence, chronic disease, sedative medication and pain. | | |
| Methods | | | | | | | |  | | |  |
| Study design | 4 | | Present key elements of study design early in the paper | | 2 | | observational cross-sectional study | | |  |  |
| Setting | 5 | | Describe the setting, locations, and relevant dates, including periods of recruitment, exposure, follow-up, and data collection | | 5 | | Recruitment and data collection was done in one long-term care institution in Switzerland  between August and December 2017. | | |  |  |
| Participants | 6 | | *Cross-sectional study*—Give the eligibility criteria, and the sources and methods of selection of participants | | 5/6 | | Recruitment targeted older adults, aged 65 years and over.  Inclusion criteria were a) ability to understand study content and sign informed consent, c) no severely impaired decision making (Cognitive performance scale > 4 points) [27].  Exclusion criteria were a) history of acute lower limb pathology (fracture and/or surgery within the last 6 months), b) skin disorders involving the anterior thigh and/or arm, c) limb paralysis and d) confinement to bed.  Residents of one nursing-home in Switzerland were screened by the senior nurse based on RAI information for inclusion and exclusion criteria. | | |  |  |
| Variables | 7 | | Clearly define all outcomes, exposures, predictors, potential confounders, and effect modifiers. Give diagnostic criteria, if applicable | | 7-10 | | Outcome: ADL performance (bed mobility, transfer, walking in a room, walking in a corridor, locomotion on the ward, locomotion outside the ward, dressing, eating/drinking, toilet use and personal hygiene over the past 7 days, each rated on a scale from 0 (independent) to 4 (fully dependent), full range of possible outcome 0-40. Categorization as independent in ADL when total score = 0, reflecting no need for assistance or staff oversight, categorization as dependent when ≥ 1, reflecting any need for assistance or staff oversight in at least one activity    Predictors: isometric muscle strength of elbow flexors and quadriceps, handgrip strength, muscle thickness and echo intensity of biceps bracchi and rectus femoris, functional mobility as assessed by the five sit-to-stand test, physical activity  Potential confounders: age, height, weight, urinary incontinence, pain intensity, falls, cognitive performance, frequency of depressive symptoms, amount and type of chronic diseases, regular intake of medication | | |  |  |
| Data sources/ measurement | 8* | | For each variable of interest, give sources of data and details of methods of assessment (measurement). Describe comparability of assessment methods if there is more than one group | | 7-10 | | Self-performance in activities of daily living (ADL) was observed, and demographics, medical and medication history were evaluated as part of the Resident Assessment Instrument by trained nurses.  One investigator, a physiotherapist, trained and experienced in musculoskeletal assessments completed the test series of muscle strength, morphology, function and physical activity for all participants:  Muscle strength  Maximal isometric quadriceps and elbow-flexor muscle strength, as the highest of two trials, was evaluated using a hand-held dynamometer (Microfet2®, CompuFET, Hoggan Health Industries, Biometrics Europe). For measurement of elbow-flexor strength, the participant was seated on a chair, elbow flexed at 90°and forearm supinated. For measurement of quadriceps strength, the participant was seated on a plinth, with their back resting against a firm support, thighs fully supported, knees flexed to 90° and the lower legs hanging freely. The curved transducer pad of a hand-held dynamometer was positioned at 80% of the forearm and tibial length respectively, to resist maximal isometric force of the elbow flexors and quadriceps. The participants were asked to push against the dynamometer as hard as possible for 3 seconds. Strength was measured in Newtons (N), and converted into Kilograms (kg) by dividing N by 9.81. Torque was calculated in Newtonmeters (Nm) by multiplying force by the lever arm length of the forearm and tibia respectively. Hand-held dynamometry has been shown to be a valid and reliable technique to assess isometric strength in older adults [29, 30]. Test-retest reliability of hand-held dynamometry is high when assessed by a trained examiner using a standardized protocol [31]; with intra-rater reliability Intraclass Correlation Coefficient (ICC) ranging between 0.90 and 0.98 [29].  Handgrip strength was measured with a hand dynamometer (Jamar®, Lafayette, USA) according to the standardized protocol recommended by the American Society of hand therapists [32].  Muscle morphology  Real-time, B-mode ultrasonography (Nemio MX Type SSA-590A, Toshiba, Japan) with a 12 MHz linear transducer array (45 mm footprint) was used to obtain transverse images of the dominant extremities. Images of the rectus femoris/vastus intermedius as well as the biceps brachii/brachialis were taken using a previously published protocol for community-dwelling older adults [33, 34], and post-processed using semi-automated MATLAB code (MathWorks®, Massachusetts, USA). Muscle thickness and primary muscle echotexture statistics were subsequently calculated, with the mean of two images taken for further analysis.  The thickness of the muscles was defined as the distance between the inner border of the fascial layer that distinguishes muscles from superficial fat and bone. Ultrasound-based measures of thigh tissue thickness are highly correlated with the gold standard of Magnetic Resonance Imaging (r=0.99) [35] and have a reported intra-rater reliability of ICC 0.88-.099 in older people [33, 36].  For grayscale analysis, settings of the ultrasound scanner (gain, time gain control, dynamic range value, focus and power) were adjusted to assure good quality of the images and kept constant for all participants; depth was readjusted to individual muscle thickness. Echogenicity of rectus femoris and biceps brachii was defined as the average grayscale within a rectangular region of interest and recorded as unspecified units (UU) 0-255. The analysis method has been previously shown to have high intra-rater reliability, with a reported ICC of 0.97-0.99 [37]. High muscle echo intensity is associated with low tissue density in CT scans [38] and high adipose tissue content in muscle biopsies [39].  Functional mobility  Functional mobility was estimated using the sit-to-stand task repeated 5 times [40]. A chair with a straight backrest, 40 cm seat height and without armrests was placed against a solid support. Participants were instructed to complete 5 sit-to-stand maneuvers as fast as possible without the use of their arms. The time taken for completion was recorded. Times in excess of 13.6 seconds are associated with increased disability [41].  Physical activity  The German physical activity 50+ questionnaire was used to calculate energy expenditure (kcal/week) [42]. The questionnaire has a test-retest reliability of r=0.52-0.6 [42] and is widely used in German speaking countries to evaluate physical activity in older people.  Frailty  Physical frailty was evaluated according to Fried’s frailty criteria [43]. Participants were characterized as “not frail”, “pre-frail” or “frail” according to the number of positive criteria identified (0, 1-2 and ≥ 3, respectively). | | |  |  |
| Bias | 9 | | Describe any efforts to address potential sources of bias | |  | | Sample size was detected by power calculation to ensure internal and external validity. Screening was done by one person. Of the residents screened, all potential participants were given study information, all were invited to participate to avoid selection bias.  Standardised protocols for assessments and valid test procedures/devices with high intra-rater and inter-rater reliability were used, assessors were all trained and experienced in relevant tests, in case of muscle tests, one investigator completed all assessments for every participant to avoid information bias.  Potential confounding variables were included in the regression analysis. | | |  |  |
| Study size | 10 | | Explain how the study size was arrived at | | 6 | | Power analysis was used to calculate the sample size based on previously published data of similar study content (investigation on relationships between muscle capacity and physical disability). After screening the nursing home residents for inclusion, all potential participants were informed about study content and invited for participation. Residents who were willing and able to sign informed consent were included ( n = 30). | | |  |  |

Continued on next page

| Quantitative variables | 11 | Explain how quantitative variables were handled in the analyses. If applicable, describe which groupings were chosen and why |  | ADL performance was categorized in two groups based on the participants’ dependence in performing activities in daily living (independent vs dependent). The aim was to evaluate if the groups differ in muscle characteristics. Dependent on data distribution, descriptive statistics are presented as mean ± standard deviation or median (range). | |
| --- | --- | --- | --- | --- | --- |
| Statistical methods | 12 | (*a*) Describe all statistical methods, including those used to control for confounding | 11 | The Statistical Package for the Social Sciences (SPSS Statistics, Version 23.0. IBM Corporation, Armonk, NY) was used for analysis. Shapiro-Wilk test was used to test for normality. Differences between the ADL groups were analyzed using independent t-tests and Mann-Whitney-U-test. Relationships of ADL performance with independent variables were identified by Spearman’s correlation coefficients and binary logistic regression analysis. Receiver-Operating Characteristic (ROC) curve was utilized for sensitivity/specificity analysis of predictors of ADL performance. | |
|  |  | (*b*) Describe any methods used to examine subgroups and interactions | 14 | Logistic regression included potential confounding factors and therefore accounted for interactions. Subgroups were not relevant. | |
|  |  | (*c*) Explain how missing data were addressed | 11/12 | Data of the five sit-to-stand test who were missing because only half of the group could complete the test were converted from interval data into ordinal data by categorization in two groups (able to complete the test, unable to complete the test). Otherwise, pairwise deletion was used in statistical analysis. | |
|  |  | (*d*) *Cross-sectional study*—If applicable, describe analytical methods taking account of sampling strategy |  | n/a | |
|  |  | (*e*) Describe any sensitivity analyses | 16 | Receiver-Operating Characteristic (ROC) curve was utilized for sensitivity/specificity analysis of predictors of ADL performance. | |
| Results | | | | |  |
| Participants | 13* | (a) Report numbers of individuals at each stage of study—eg numbers potentially eligible, examined for eligibility, confirmed eligible, included in the study, completing follow-up, and analysed |  | Potentially eligible and examined for eligibility n=177, confirmed eligible, included and analysed n=30 | |
|  |  | (b) Give reasons for non-participation at each stage |  | Of 177 residents potentially eligible for participation, 147 were excluded due to the following reasons: inability or unwilling to sign informed consent, acute deterioration of health condition | |
|  |  | (c) Consider use of a flow diagram |  | n/a | |
| Descriptive data | 14* | (a) Give characteristics of study participants (eg demographic, clinical, social) and information on exposures and potential confounders | 12/13 | Participants’ characteristics of the ADL dependent and independent group (item of medical and medication history were considered as potential confounders)   \| Characteristic  (unit) \| ADL independent \| ADL dependent \| equality of means/ medians \| \| \| --- \| --- \| --- \| --- \| --- \| \|  \| Mean (SD)/  Median (range) \| Mean (SD)/  Median  (range) \| t/U \| p \| \| **Demographics** \|  \|  \|  \|  \| \| *Age^†^ (years)* \| 87.0 (15) \| 86.0 (35) \| U = 100.5 \| .64 \| \| *Weight^†^ (kg)* \| 66.5 (52.2) \| 68.0 (51) \| U = 98.0 \| .58 \| \| *Height^†^ (m)* \| 1.64 (0.23) \| 1.59 (0.21) \| U = 80.5 \| .19 \| \| *Incontinence (0-4 points)* \| 0 (1) \| 0 (4) \| U = 68 \| .038* \| \| *Pain intensity (0-10)* \| 0.53 (0.74) \| 0.64 (1.60) \| t(18.1) = -.234 \| .82 \| \| *Falls (number)* \| 0.63 (1.63) \| 0.29 (0.47) \| t(17.8) = .797 \| .44 \| \| *Cognitive performance (0-6)* \| 1.0 (3) \| 2.0 (3) \| U = 79 \| .24 \| \| *Depressive symptoms (0-32)* \| 1.62 (3.95) \| 0.71 (1.27) \| t(25) = 0.815 \| .42 \| \| *Chronic diseases (number)* \| 2.75 (1.24) \| 3.86 (1.29) \| t(27.1) = -2.387 \| .024* \| \| *Medication (number)* \| 0.81 (0.40) \| 0.79 (0.43) \| t(27.0) = .176 \| .86 \| \| *Institutionalization (years)* \| 2.9 (2.2) \| 3.4 (1.8) \| t(27.8) = -.572 \| .57 \| \| **Muscle strength** \|  \|  \|  \|  \| \| *Handgrip strength^†^ (kg)* \| 16.0 (22) \| 13.3 (13) \| U = 63.0 \| .043* \| \| *Quadriceps strength^†^ (kg)* \| 13.1 (5.7) \| 8.8 (6.9) \| U = 18.5 \| <.001* \| \| *Quadriceps strength^†^ (N)* \| 128.2 (56.4) \| 85.4 (63.3) \| U = 18.5 \| <.001* \| \| *Strength/body weight (N/kg)* \| 2.1 (0.7) \| 1.4 (0.4) \| t(16.7) = 2.797 \| .013* \| \| *Quadriceps torque^†^ (Nm)* \| 39.1 (18.8) \| 26.8 (20.3) \| U = 20 \| .001* \| \| *Torque/body weight (Nm/kg)* \| 0.6 (0.2) \| 0.4 (0.1) \| t(16.9) = 2.763 \| .013* \| \| *Elbow-flexor strength^†^ (kg)* \| 9.7 (7.3) \| 7.0 (9.5) \| U = 43.0 \| .036* \| \| **Muscle morphology** \|  \|  \|  \|  \| \| *Quadriceps thickness (mm)* \| 17.7 (4.7) \| 18.7 (6.7) \| t(28) = -.465 \| .65 \| \| *Rectus femoris grayscale^†^ (UU)* \| 117.2 (78) \| 115.5 (101) \| U = 109.0 \| .92 \| \| *Elbow-flexor thickness^†^ (mm)* \| 24.8 (18) \| 26.5 (16) \| U = 111 \| .98 \| \| *Biceps brachii grayscale^†^ (UU)* \| 132 (81) \| 133 (86) \| U = 111 \| .98 \| \| **Functional mobility** \|  \|  \|  \|  \| \| *5 sit-to-stand (sec)* \| 20.3 (11.6) \| 20.6 (12.3) \| t(8.0) = -.051 \| .96 \| \| **Physical activity (kcal/week)** \| 842 (879) \| 508 (1347) \| t(21.9) = .792 \| .44 \| | |
|  |  | (b) Indicate number of participants with missing data for each variable of interest |  | Four participants were excluded from strength measurements due to concerns associated with the risk of osteoporotic fracture and 14 were incapable of rising from a chair without using their arms. | |
|  |  | *c) Cross-sectional study—*Report numbers of outcome events or summary measures |  | The one outcome event is ADL performance. 16 were categorized as independent and 14 as dependent in ADL. | |
| Main results | 16 | (*a*) Give unadjusted estimates and, if applicable, confounder-adjusted estimates and their precision (eg, 95% confidence interval). Make clear which confounders were adjusted for and why they were included | App1-3 | Correlations of ADL category with demographics, medical and medications history   \| variable \| Correlation coefficient (r_s_) \| Significance (p) \| \| --- \| --- \| --- \| \| age \| -.09 \| .639 \| \| sex \| -.12 \| .542 \| \| height \| -.24 \| .194 \| \| weight \| .11 \| .570 \| \| urinary incontinence \| .39 \| .037* \| \| pain intensity \| -.12 \| .543 \| \| falls \| .07 \| .701 \| \| cognitive performance \| .23 \| .238 \| \| frequency of depressive symptoms \| .09 \| .660 \| \| symptoms of physical frailty \| .22 \| .234 \| \| amount of chronic diseases \| .41 \| .027* \| \| regular intake of medication \| -.03 \| .861 \|   *significant difference  Correlations of ADL category with muscle strength, muscle morphology, functional mobility and physical activity   \| variable \| Correlation coefficient (r_s_) \| Significance (p) \| \| --- \| --- \| --- \| \| muscle strength \|  \|  \| \| handgrip strength \| -0.38 \| =.038* \| \| elbow flexor strength \| -0.42 \| =.032* \| \| quadriceps strength \| -0.67 \| <.001* \| \| muscle morphology \|  \|  \| \| quadriceps thickness \| .01 \| =.968 \| \| rectus femoris grayscale \| -.02 \| =.903 \| \| elbow-flexor thickness \| -.01 \| =.968 \| \| biceps brachii grayscale \| .01 \| =.968 \| \| functional mobility/ 5 sit-to-stand category \| -.21 \| =.276 \| \| physical activity \| -0.44 \| =.015* \|   *significant difference  Binary logistic regression with inclusion analysis of the variables quadriceps strength, chronic diseases and physical activity   \| predictor \| Regression coefficient (B) \| Significance level (p) \| Exp(B)=OR \| 95% CI for Exp(B) \| \| \| --- \| --- \| --- \| --- \| --- \| --- \| \|  \|  \|  \|  \| lower \| upper \| \| quadriceps strength \| -1.040 \| .030 \| 0.353 \| 0.138 \| 0.905 \| \| chronic diseases \| 1.325 \| .074 \| 3.763 \| 0.877 \| 16.139 \| \| handgrip strength \| -.213 \| .390 \| 0.809 \| 0.498 \| 1.313 \| \| constant \| 10.863 \| .067 \| 52183.975 \|  \|  \| | |
|  |  | (*b*) Report category boundaries when continuous variables were categorized | 8, 11 | Category boundary of ADL performance:  The performance of 10 activities of daily living was observed, each rated on a scale from 0 (independent) to 4 (fully dependent), full range of possible outcome 0-40.  Categorization as independent in ADL when total score = 0, reflecting no need for assistance or staff oversight, categorization as dependent when ≥ 1, reflecting any need for assistance or staff oversight in at least one activity.  Given the fact that only half of the participants could rise from a chair 5 times without using their arms, the variable was dichotomized into the categories “able to complete 5 STS” and “unable to complete 5STS” for further analysis. | |
|  |  | (*c*) If relevant, consider translating estimates of relative risk into absolute risk for a meaningful time period |  | n/a | |

Continued on next page

| Other analyses | 17 | Report other analyses done—eg analyses of subgroups and interactions, and sensitivity analyses |  | The ROC curve for analysis of sensitivity and specificity of quadriceps strength to identify people independent and dependent in ADL showed an area under the curve of 0.89. Strength of 11.25 kg was the best fitting value with a sensitivity of 100% and a specificity of 79%. |
| --- | --- | --- | --- | --- |
| Discussion | | | | |
| Key results | 18 | Summarise key results with reference to study objectives | 17,19 | Of the investigated parameters, greater handgrip-strength, elbow-flexor strength, quadriceps strength and physical activity, as well as less incontinence and chronic diseases, were positively associated with the ability to independently perform basic ADL whereas quadriceps strength was the only independent predictor. Isometric quadriceps strength of > 11kg predicted ADL-independence by 100% and ADL-dependence by 79% in this study sample. |
| Limitations | 19 | Discuss limitations of the study, taking into account sources of potential bias or imprecision. Discuss both direction and magnitude of any potential bias | 20/21 | Firstly, observations by nursing staff of behavior and emotions by nursing staff entailed the risk that some actions of the participants could have been unrecognized and therefore not recorded adequately. However, the observation period included seven days in which residents were closely observed by a trained nurse attentive to precise assessment. Therefore, the risk of information bias was likely to be small. Even though RAI data were obtained by different assessors, data can still be assumed sufficiently reliable since inter-rater reliability of the RAI items has been shown to be 0.63-0.92 (weighted Kappa) [60]. Secondly, physical activity measures were based on self-report and behavioral observations of nursing staff. Although previous studies have questioned the accuracy of self-reported measures of physical activity in older populations [61], participants were closely monitored during the study so that any discrepancy between reported and actual activity was likely to be small. Thirdly, the number of participants who could complete the timed sit-to-stand task was rather small. Findings with regard to this variable could therefore be underpowered. However, even when the participants were categorized into two groups depending on their ability to complete the task, results did not change. Therefore, the results were assumed to be valid for this cohort. Fourthly, a causal relationship between ADL performance and quadriceps strength cannot be made due to the nature of the cross-sectional study design. However, the results indicate a strong association between ADL dependence and low quadriceps strength and longitudinal studies have demonstrated beneficial effects of quadriceps’ strength training on physical function [21-26]. Fifthly, the study sample only included participants from one nursing home. Therefore, the results of the sample might not be generalizable to a wider population of older, frail nursing home residents. The study did, however, include a wide variety of participants with regard to ADL performance. Therefore, the participants could be considered representative of the target population. |
| Interpretation | 20 | Give a cautious overall interpretation of results considering objectives, limitations, multiplicity of analyses, results from similar studies, and other relevant evidence | 21 | This study has shown that strength, physical activity and incontinence were potentially modifiable factors associated with ADL dependence in nursing home residents, with quadriceps strength being the only independent predictor of dependence in ADL, independent of age, frailty status, co-morbidities and cognitive function. Although further research is required, interventions aimed at increasing these physical abilities with a specific focus on enhancing leg muscle strength beyond target threshold values may be a useful strategy for reducing dependence in ADL of nursing home dwellers. |
| Generalisability | 21 | Discuss the generalisability (external validity) of the study results | 21 | The study sample included only participants of one nursing home. Therefore, the results of the sample might not be generalizable to the population of old, frail nursing home residents. After all, the study included a great variety of participants in regard to ADL performance, therefore, the participants could be considered representative for the target population. |
| Other information | |  | | |
| Funding | 22 | Give the source of funding and the role of the funders for the present study and, if applicable, for the original study on which the present article is based |  | No funding |

*Give information separately for cases and controls in case-control studies and, if applicable, for exposed and unexposed groups in cohort and cross-sectional studies.

**Note:** An Explanation and Elaboration article discusses each checklist item and gives methodological background and published examples of transparent reporting. The STROBE checklist is best used in conjunction with this article (freely available on the Web sites of PLoS Medicine at http://www.plosmedicine.org/, Annals of Internal Medicine at http://www.annals.org/, and Epidemiology at http://www.epidem.com/). Information on the STROBE Initiative is available at www.strobe-statement.org.
